# An in-depth analysis of the prognostic significance and potential clinical impact of Leupaxin in the immunotherapeutic treatment of esophageal squamous cell carcinoma

**DOI:** 10.1016/j.gendis.2025.101695

**Published:** 2025-05-27

**Authors:** Fei Teng, Yang Chen, Xiaojuan Zhang, Hai-Ke Lei, Mei He, Wei Wang, Zhi-Qiang Wang, Gui-Xue Wang

**Affiliations:** a Key Laboratory for Biorheological Science and Technology of Ministry of Education (Chongqing University), Chongqing University Cancer Hospital, Chongqing 400030, China; b Key Laboratory for Biorheological Science and Technology of Ministry of Education, State and Local Joint Engineering Lab for Vascular Implants, College of Bioengineering, Chongqing University, Chongqing 400044, China

Esophageal cancer (EC), with annual global reports exceeding 570,000 fresh cases, presents a relatively prevalent concern.^1^ Esophageal squamous cell carcinoma (ESCC), the most prevalent histological subtype of EC, is particularly common in southern Africa and southern Asia. It constitutes 90% of EC cases in China. This disease, characterized by aggressive tumor growth, significant tumor heterogeneity, and complex oncogenic pathways, typically has a poor prognosis. Recent years have seen considerable advancements in cancer immunotherapy with the advent of immune checkpoint inhibitors (ICIs) such as cytotoxic T-lymphocyte-associated protein 4 (CTLA-4) and programmed cell death protein 1 (PD-1).^2^ Nonetheless, the absence of effective treatment strategies to surmount resistance to cancer immunotherapy precludes a large number of cancer patients from reaping its benefits or achieving enduring therapeutic results.^3^ Identifying new immunotherapy biomarkers or novel immunoregulatory genes could pave the way for a more tailored and durable cancer immunotherapy approach. Elevated levels of Leupaxin (LPXN) have been correlated with the evolution and progression of certain malignant tumors,^4^ such as prostate cancer, colon cancer, breast cancer, and osteosarcoma. However, the impact of LPXN on the progression, prognosis, and its immunomodulatory functions in ESCC remains unknown.

In our study, we first used Gene Expression Profiling Interactive Analysis (GEPIA) and UALCAN Omnibus to analyze the expression levels of LPXN in EC/ESCC. We found that LPXN showed higher expression in EC/ESCC compared to the healthy control group (Fig. 1A, B). Concurrently, using the ‘survival’ tool in R, we analyzed the ESCC dataset GSE53625. By calculating the optimal cutoff value, LPXN expression was categorized into high and low groups. Furthermore, the Kaplan–Meier survival analysis highlighted a significant correlation, where greater LPXN expression was associated with a poorer overall survival rate (hazard ratio = 0.78, p = 0.015) (Fig. 1C). We collected 10 pairs of clinical samples of esophageal squamous cell carcinoma from Chongqing University Cancer Hospital to verify the expression of LPXN. Both Immuno-Histochemical (IHC) and Quantitative Real-Time PCR (q-PCR) analyses indicated that the expression of LPXN aligned consistently with the RNA sequencing datasets from both GEPIA and UALCAN (Fig. 1D‒F). The percentage of positive cells in the tumors for each patient are presented in Table S1.This indicates that LPXN could be a potential oncogene for ESCC.

**Figure 1:**
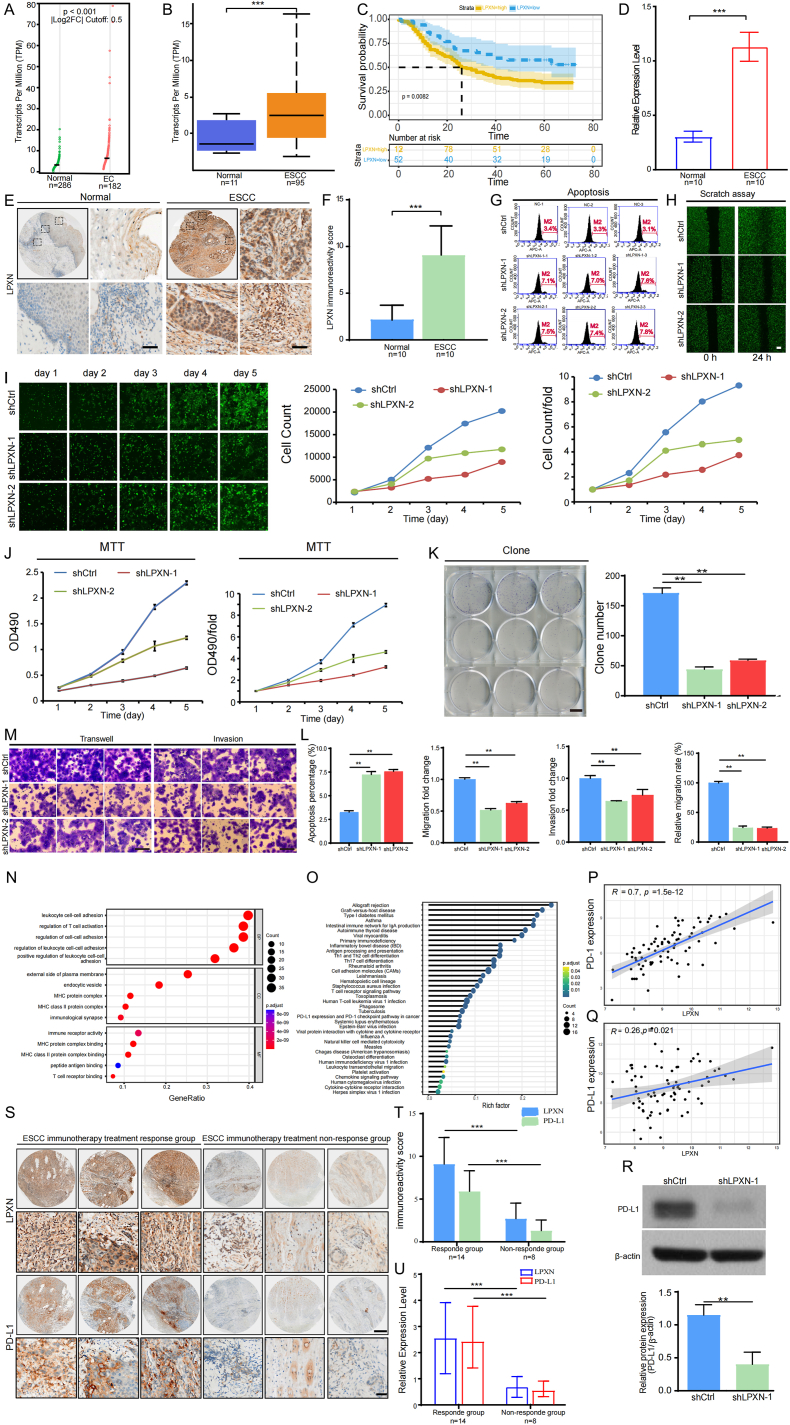
LPXN promotes the occurrence and progression of esophageal squamous cell carcinoma and its clinical relevance to immunotherapy efficacy. **(A)** Scatter plots show upregulated LPXN levels in EC tissues compared with the level in normal tissues (*p* < 0.001) using the Gene Expression Profiling Interactive Analysis tool (GEPIA). **(B)** The overexpression of LPXN was validated in ESCC compared with the level in normal tissues using the UALCAN omnibus (∗∗∗*p* < 0.001). **(C)** Overall survival analysis indicates that the high-expression group of LPXN has a poorer prognosis than the low-expression group (*p* = 0.008). **(D)** q-PCR was performed on ten pairs of ESCC tissues and normal tissues to validate the mRNA-level overexpression of LPXN in ESCC tissues (∗∗∗*p* < 0.001). **(E)** immunohistochemistry (IHC) for LPXN was performed on ten pairs of ESCC tissues and normal tissues to confirm the overexpression of LPXN at the protein level in ESCC samples, with low magnification at × 50 and high magnification at × 500 (Scale bar = 100 μm). **(F)** The range of LPXN expression scores in ten matched pairs of ESCC tissues are visualized using a box plot (∗∗∗*p* < 0.001). **(G)** Flow cytometry analysis of apoptosis and the cell cycle indicated that, compared to the control group, the proportion of apoptotic tumor cells increased after the expression of LPXN was knocked down. **(H)** Celigo detected a decrease in the migratory ability of KYSE-150 cells in the experimental group 24 h after scratch assay. **(I)** Celigo was used for detection for 5 days continuously; the proliferation rate of experimental group KYSE-150 cells was significantly inhibited. **(J)** MTT assay revealed significant inhibition of the proliferation rate of KYSE-150 cells in the experimental group. **(K)** Cloning experiment revealed a reduction in the colony number of KYSE-150 cells in the experimental group. **(M)**The migration (Transwell without extracellular matrix (ECM)) and the invasion (Transwell with ECM) assay revealed significant inhibition of the metastatic ability of KYSE-150 cells in the experimental group. **(L)**We quantified and represented the apoptosis percentage, migration fold change, invasion fold change, and relative migration rate between the experimental group and the control group using box plots (∗∗*p* < 0.01). **(N)**Gene Ontology (GO) enrichment analysis of LPXN in ESCC. Functions are categorised as biological processes (BP), cellular components (CC), and molecular functions (MF). (**O**)the Kyoto Encyclopedia of Genes and Genomes (KEGG) enrichment analysis of LPXN in ESCC. **(P, Q)** Correlation analysis of LPXN with PD1 expression (*p* < 0.001), PD-L1 expression (*p* = 0.021) in ESCC. **(R)** Using Western blot analysis, the expression of PD-L1 was significantly reduced in both the shLPXN-1 and shLPXN-2 groups compared to the shCtrl group. Additionally, a box chart was generated to compare the relative expression levels of PD-L1 protein to β-actin between the knocked-down KYSE-150 cells and the control cell line, respectively (∗*p* < 0.05, ∗∗*p* < 0.01). **(S)** Immunohistochemical (IHC) analysis was conducted on ESCC tissues with different immunotherapy efficacies to validate the correlation between the expression of LPXN and PD-L1 at the protein level, as well as the efficacy of immunotherapy (low magnification × 50; high magnification × 500). **(T)** The expression scores of LPXN and PD-L1 in ESCC tissues with different immunotherapy efficacies are visualized using a box plot (∗∗∗*p* < 0.001). **(U)** Quantitative PCR (q-PCR) was performed on ESCC tissues with different immunotherapy efficacies to validate the correlation between the expression of LPXN and PD-L1 at the mRNA level, as well as the efficacy of immunotherapy. The results were presented using a box plot (∗∗∗*p* < 0.001).

Furthermore, when we knocked down LPXN in KYSE-150 cells (Fig. S1A, B), it resulted in a significant decrease in the number of KYSE-150 cells, an increase in apoptosis, and a significant reduction in migration speed. These findings were evidenced by the flow cytometry, scratch tests, Celigo assay, MTT assay, cell cloning, transwell migration and transwell invasion (Fig. 1 G‒L). These findings suggest that LPXN Serves as fundamental role in cellular growth, proliferation, and migration. To explore the molecular underpinnings of LPXN in the pathogenesis and development of ESCC, we performed functional enrichment analysis on LPXN. We utilized the data from The Cancer Genome Atlas (TCGA) to perform Gene Ontology (GO) and Kyoto Encyclopedia of Genes and Genomes (KEGG) analyses on a cohort of eighty-eight genes that are co-expressed with LPXN. The results were then visualized using R software. According to the GO analysis results, LPXN modulates the molecular functions of immune receptors, influences the cellular compartments at the immunological synapse, and impacts the biological process of T cell activation (Fig. 1N). The pivotal immune-related pathways elucidated in the KEGG analysis included Th1 and Th2 cell differentiation, the T-cell receptor signaling pathway, and the cancer-related expression of PD-L1 and PD-1 checkpoint pathways (Fig. 1O). These findings indicate that LPXN might be involved in various immunoregulatory mechanisms in ESCC and promote its occurrence.

The PD-1/PD-L1 pathway is crucial in inhibiting cancer cells from circumventing the immune response and disseminating throughout the body.^5^ Personalized immunotherapy necessitates the assessment of immunological checkpoints, including PD-1 and PD-L1. Our analysis revealed a significant correlation between the expression levels of PD-1 and PD-L1 and the expression values of LPXN derived from the TCGA database (Fig. 1P, Q; *p* = 1.5∗10^−12^ and *p* = 0.021, respectively). Since immunohistochemical protein expression of PD-L1 is the most important predictive indicator of immunotherapy efficacy in malignant tumors clinically, we validated the relationship between LPXN and PD-L1 expression at the protein level through western blotting analysis. Our results demonstrated that, relative to the cells transfected with the empty vector, the protein expression levels of PD-L1 were diminished in the LPXN knockdown cell lines (Fig. 1R). These observations imply a direct correlation between LPXN expression and PD-L1 upregulation, corroborating the findings derived from the TCGA dataset.

To substantiate the relationship between LPXN expression and the therapeutic outcomes of immunotherapy in a clinical setting, we procured tumor specimens from 22 patients who received immunotherapy at the Chongqing University Cancer Hospital. The clinical characteristics of ESCC samples with diverse immunotherapy responses are detailed in Table S2. q-PCR and immunohistochemical (IHC) staining analyses demonstrated that both LPXN and PD-L1 were upregulated in tumor samples from patients exhibiting a favorable treatment response compared to those with a poor response (Fig. 1S, T, U). This suggests that LPXN and PD-L1 are concurrently overexpressed in ESCC and are indicative of a positive response to immunotherapy. These data enable us to infer the potential efficacy of immunotherapy in ESCC and deduce PD-L1 expression levels based on the expression of LPXN.

In conclusion, through a combination of bioinformatics analysis and molecular and cellular tests, this study has demonstrated that LPXN is highly expressed in ESCC. Furthermore, the expression of LPXN is directly proportional to the malignancy of ESCC. Clinical samples were collected to validate the high expression of LPXN in ESCC. Additionally, we explored the correlation between LPXN and tumor immunity. An enrichment analysis of GO and KEGG on samples of ESCC from the TCGA database revealed that LPXN might influence the progression of ESCC by participating in various immune pathways. Thus, through cellular experiments, the positive correlation between LPXN and PD-L1 was validated at both the mRNA and protein levels. To further our understanding, we collected specimens of ESCC post-immunotherapy from clinical practice. Based on different effects of immunotherapy, it was observed that LPXN and PD-L1 show distinctly high expressions in the effective response group (Table S3 lists the principal antibodies for LPXN and PD-L1). Consequently, we postulate that LPXN is a significant potential biomarker for ESCC. The direct correlation with PD-L1 expression can indirectly predict the therapeutic effect of immunotherapy on ESCC.

## CRediT authorship contribution statement

**Fei Teng:** Methodology, Writing – original draft, Writing – review & editing. **Yang Chen:** Funding acquisition, Methodology, Writing – original draft, Writing – review & editing. **Xiaojuan Zhang:** Methodology, Writing – original draft. **Hai-Ke Lei:** Formal analysis, Writing – review & editing. **Mei He:** Writing – original draft. **Wei Wang:** Formal analysis. **Zhi-Qiang Wang:** Conceptualization, Funding acquisition, Writing – review & editing. **Gui-Xue Wang:** Conceptualization, Funding acquisition, Writing – review & editing.

## Availability of data and materials

The datasets analyzed for this study are accessible from the corresponding author upon submission of a reasonable request.

## Ethics approval

This research was carried out in compliance with the Declaration of Helsinki, as revised in 2013. The Ethics Committee of Chongqing University Cancer Hospital granted approval for the study (CZLS2024141-A).

## Consent for publication

Informed consent was obtained in writing from all participants enrolled in this study, and they have consented to the dissemination of their research outcomes.

## Funding

This work was supported by the Natural Science Foundation of Chongqing (CSTB2024NSCQ-KJFZMSX0105), the Natural Science Foundation of Chongqing (CSTB2024NSCQ-KJFZZDX0011) and the Postdoctoral Science Foundation of China (No. 2023M730435). The Rapid Service Fee was funded by the authors.

## Conflict of interests

The authors certify that there are no conflict of interests to disclose.

## Figures and Tables

**Fig. 1:**
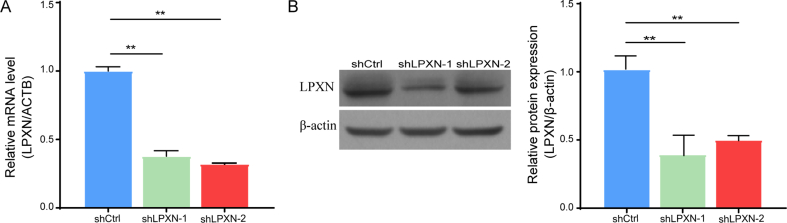
In KYSE-150 cells, after knocking down LPXN, the expression of LPXN at both mRNA and protein levels. **(A)** q-PCR detects the inhibition of LPXN gene expression at the mRNA level in the experimental group KYSE-150 cells. (**B)** The Western blot analysis demonstrated a substantial decrease in the protein expression of the targeted LPXN gene at the endogenous level in KYSE-150 cells following knockdown.

## Appendix A Supplementary data

Supplementary Data 1 Materials and Methods. Supplementary Data 1

Supplementary Table 1 The percentage of positive cells (positive cells/total cells). Supplementary Table 1

Supplementary Table 2 Clinical information of 32 patients providing tissue samples. Supplementary Table 2

Supplementary Table 3 The information of primary antibodies used in IHC. Supplementary Table 3

## References

[1] Guo X., Gao C.Y., Yang D.H., Li S.L. (2023). Exosomal circular RNAs: a chief culprit in cancer chemotherapy resistance. Drug Resist Updat.

[2] Poggio M., Hu T.Y., Pai C.C. (2019). Suppression of exosomal PD-L1 induces systemic anti-tumor immunity and memory. Cell.

[3] Sun Y., Revach O.Y., Anderson S. (2023). Targeting TBK1 to overcome resistance to cancer immunotherapy. Nature.

[4] Khan A., Li W.F., Ambreen A., Wei D.Q., Wang Y.J., Mao Y.S. (2022). A protein coupling and molecular simulation analysis of the clinical mutants of androgen receptor revealed a higher binding for Leupaxin, to increase the prostate cancer invasion and motility. Comput Biol Med.

[5] Nolan E., Lindeman G.J., Visvader J.E. (2023). Deciphering breast cancer: from biology to the clinic. Cell.

