# Supplementary material for: An in-depth analysis of the prognostic significance and potential clinical impact of Leupaxin in the immunotherapeutic treatment of esophageal squamous cell carcinoma
Source: Genes Dis. 2025 May 27;13(1):101695. doi: 10.1016/j.gendis.2025.101695 (PMC12624606; doi:10.1016/j.gendis.2025.101695)
Supplement: Supplementary Data 1 — Materials and Methods. [file mmc1.docx]

**Materials and methods**

**GEPIA**

Normal samples from the transcriptional data of 9,736 tumours and 8,587 from the Genotype-Tissue Expression projects and Cancer Genome Atlas (TCGA) were evaluated using the GEPIA web-based tool (http://gepia.cancer-pku.cn/index.html). Tumour and normal differential expression analysis, profiling by cancer type or pathological stage, patient survival analysis, dimensionality reduction analysis, comparable gene detection, and correlation analysis are features that can be modified to meet user requirements. We compared LPXN expression between esophageal malignancies and adjacent normal tissues using GEPIA. Both the fold-change and *p*-value cutoffs were set at 0.01.

**The UALCAN Omnibus**

Using clinical data from 31 different cancer types and level 3 RNA sequencing (seq-RNA) from TCGA, UALCAN was identified as an omnibus. It is an interactive and user-friendly web resource. We used UALCAN to evaluate changes in LPXN expression during the disease and to confirm that its expression in ESCC differs from that in healthy tissues.

**The GSE53625 sample from the GEO database**

GSE53625, downloaded from GEO, comprises the clinical data and RNA sequencing results of 179 cases of ESCC. The samples submitted by the published literature and used in the GSE53625 dataset were obtained from Chinese patients. These data were used to examine the value of LPXN in patients with ESCC.

**TCGA** **database**

RNA was sequenced from 80 patients with ESCC based on information retrieved from TCGA website (http://tcga-data.nci.nih.gov/tcga). As a public database, TCGA contains information on the genomic, epigenomic, transcriptomic, and proteomic characteristics of 33 different cancer types and their matched normal samples. Correlations were tested using Spearman’s correlation, and statistically significant differences were defined as *p* < 0.05 and > 0.7. Based on the genes with expressions highly linked with LPXN expression, GO and KEGG analysis were conducted using the ‘clusterProfiler’ and ‘KEGG.db’ R packages, respectively.

The scores of stromal and immunological in each ESCC sample in TCGA database were determined by enrichment analysis using a gene set of a single sample (ssGSEA) and the ‘GSVA’ R package. Stromal cells and immune infiltration in distinct tumour tissues were evaluated using these scores. We investigated the relationship between LPXN expression and ratings using the Spearman correlation test. Using features gleaned from the transcriptome and epigenetics of tumour samples from TCGA, stem cell-like properties of tumours can be quantified. The link between the infiltrating immune subtype and LPXN expression was investigated using one-way analysis of variance (ANOVA). Using Spearman’s correlation, the relationships between LPXN and PD-L1/PD1, MRP, and 47 widely utilised immune checkpoint genes were assessed. Statistical significance was set at *p* < 0.05.

**Quantitative polymerase chain reaction (q-PCR)**

Twenty samples were obtained from the Chongqing University Cancer Hospital, including ten pairs of tumour and healthy esophageal tissues, 14 immune-responsive ESCC tissues, and eight immune-resistant ESCC tissues. Twenty-two patients with ESCC who had been treated with adjuvant immune therapy were included. Nanoparticle albumin-bound paclitaxel, cisplatin, and pembrolizumab were used in a four-cycle therapy plan for patients with ESCC in compliance with the 2021 Chinese Society for Clinical Oncology guidelines. Patients with ESCC had their responses to immune treatment measured based on the iRECIST criteria. The Hospital ethics board approved this study. We analysed the expression of LPXN in ESCC and normal esophageal tissues. We further analysed immune-resistant ESCC tissues and LPXN expression using q-PCR. RNA from each sample was acquired independently using TRIzol (Servicebio) according to the manufacturer's instructions. Using a Thermo Revert Aid First Strand cDNA Synthesis Kit and reverse transcription, RNA was converted to cDNA. Step One Plus (Applied Biosystems) q-PCR analysis was performed using the Fast Start Universal SYBR Green Master Mix (Roche). The 2Ct technique was used for data analysis, and the endogenous control, GAPDH, was measured. The primer sequences for LPXN and ATCB as follows: LPXN forward, 5'-CCACCACCTTCTAAAACGTCAG-3', reverse, 5'-CCCAAGCATTGAGTCCAG-GG-3'; ATCB forward5'-GCGTGACATTAAGGAGAAGC-3', reverse, 5'- CCACG-TCACACTTCATGATGG-3'.

**Immunohistochemistry (IHC) staining**

42 clinical samples were obtained from Chongqing University Cancer Hospital, including 10 pairs of ESCC tissues and adjacent non-cancerous tissues, as well as 14 immune-responsive ESCC tissues and 8 immune-resistant ESCC tissues. The acquisition of these samples was approved by the Institutional Review Board of the hospital.

All specimens were immersed in paraffin, fixed in 10% formalin at 25°C, and sectioned at 4 mm serial thickness for the IHC experiment. Antigen recovery was performed by boiling the dewaxed hydrated tissue slices in 10 mmol/L citrate solution (pH 6.4) for 10 min. Using methanol, hydrogen peroxide 3% was used to inactivate endogenous peroxidase, and citric acid buffer (pH 6.0) was used to treat the slices to recover as much antigen as possible. Bovine serum albumin (1% concentration) was treated with phosphate buffer for 30 min to reduce non-specific binding. The slices were treated with primary antibody for 12 h at 4°C. The sections were then rinsed thrice in saline phosphate buffer for 5 min each. The slices were then incubated for 50 min with a 1:200 dilution of secondary antibody (Horseradish Peroxidase polymer). The samples were sealed and examined under a light microscope. The primary antibodies used were anti-LPXN (PA597933, Thermofisher), and anti-PD-L1 (PA528115, Thermofisher). Supplementary Table 3 lists the principal antibodies.

**Semi‑quantitative assessment and scoring**

To evaluate the IHC results, we followed a previously established scoring method. The expression of LPXN was assessed using a semi-quantitative approach based on the percentage of positive cells and the intensity of cytoplasmic/nuclear staining. The percentage of positive cells was categorized as follows: 0 (less than 5% positive cells), 1 (6-25% positive cells), 2 (26-50% positive cells), 3 (51-75% positive cells), or 4 (more than 75% positive cells). Cytoplasmic/nuclear staining intensity was classified as: 0 (negative), 1 (light yellow), 2 (yellow), or 3 (brown). The product of the percentage of positive cells and staining intensity yielded the Immunohistochemistry score (IS) for each patient. Two independent pathologists assessed all immunostained results and arrived at a consistent score. If there was disagreement between the two pathologists regarding the immunohistochemical results, a third pathologist was consulted for further analysis. Therefore, the IS ranged from 0 to 12, reflecting the scoring system used.

**Small hairpin RNAs (shRNAs) and lentiviruses**

The shRNA polynucleotides shLPXN-1 (CCAACGACTACCACCAACTTT) and shLPXN-2 (GCACTTCTTCTGCTCTCACTG) which target human LPXN, were synthesised, annealed, and cloned into the lentiviral vector GV493 carrying a green fluorescent protein expression cassette (GeneChem Co., Ltd., Shanghai, China). An empty GV493 vector served as the control shRNA. The models were tested using these sequences.

**Western blot analysis**

We conducted Western blot analyses using cell lysates obtained from KYSE-150 human ESCC cell lines and ESCC tissue. An equal amount of protein lysate was loaded and separated on a 10% SDS-polyacrylamide gel electrophoresis system, followed by transfer onto a PVDF membrane (Millipore, USA). Subsequently, the membranes were blocked with 5% fat-free milk in TBS buffer and incubated overnight at 4°C with the following primary antibodies: LPXN (PA597933, Thermo Fisher), PD-L1 (PA528115, Thermo Fisher), and β-actin (HRP-66009, Proteintech). Bound antibodies were detected using a secondary antibody (Horseradish Peroxidase polymer) at room temperature for 2 hours. After washing, the resulting bands were visualized using the standard Electron Cryo-Microscopy (ECL) procedure (Kangwei, Beijing). The acquired images were analyzed for grayscale values using the image acquisition system (BioRad, USA) and the ImageJ image analysis program (the National Institutes of Health, USA).

**Cell culture and transfection**

Cultures of KYSE-150 human ESCC cell lines were maintained in 6-cm plates with 3 mL of complete media in a 37°C, 5% CO_2_ incubator after being obtained from GeneChem Co., Ltd. (Shanghai, China). However, the tests were conducted using logarithmically growing cells. Transfection of shRNA (shLPXN-1, PSC107025), shRNA (shLPXN-2, PSC110829), and negative control sequence viruses was performed on KYSE-150 cells cultured in 6-well plates or 60 mm^2^ dishes using the same method as previously described for the generation of lentiviruses. The efficacy of the infection was assessed 72 h after transfection using a fluorescence microscope (IX71; Olympus, Japan). Blotting was performed using RT-PCR to analyse the mRNA and protein expression levels of LPXN in cells infected with shLPXN-1, shLPXN-2, or shCtrl to assess the effectiveness of knockdown.

**Celigo image cytometry assay**

Logarithmic-phase experimental cells were dissociated with trypsin and centrifuged to collect them. In 96-well plates, the cells were resuspended, quantified, and seeded at a density of 2 × 10^3^ cells/well in 100 μL growth medium. Seeding was performed to obtain the same number of cells from each well. The cells were cultured at 37°C and 5% CO_2_. The fluorescence intensity of the cells was measured daily for 5 consecutive days using the Celigo Imaging Cytometer System (Nexcelom Bioscience, St. Lawrence, MA, USA), and the cell number was automatically calculated. A proliferation curve was created using the number of green fluorescent cells.

**Flow cytometry analysis of apoptosis and the cell cycle**

A single-cell suspension of trypsin-digested logarithmic-phase cells that had undergone two cold PBS washes was administered to each test subject group. We adhered to the instructions provided by the manufacturer of the Annexin V-APC apoptosis detection kit (eBioscience, San Diego, CA, USA). A volume of 10 μL Annexin V-APC was used to stain the cell suspensions at room temperature for 10–15 min in the dark. A flow cytometer (BD Biosciences, San Jose, CA, USA) was used to calculate the percentage of dead cells.

**Colony forming assay**

Each cell was plated at a density of 500 in well plates for 14 days in full medium and observed every 3 days. After incubation in 4% paraformaldehyde for 30–60 min, the wells were washed with PBS to remove any remaining fixative. A crystal violet staining solution (1000 μL), filtered to remove any impurities, was used to stain the cells for 10–20 min. The cells were analysed by employing multiple ddH_2_O washes, drying at room temperature, digital photography, and colony counting. The number of visible colonies was counted using the Image-Pro Plus software (Version 6.0.0; Media Cybernetics Inc).

**MTT assay**

Trypsin was employed to dissociate the developing cells in the logarithmic phase, which were subsequently separated by centrifugation. At a density of 2 × 10³ cells per well, the cells were resuspended and plated into a 96-well plate. After a 24-hour incubation period, cells in each group were treated with sterile MTT (5 mg/mL) at 37°C for 4 hours on days 1 through 5. Following the removal of the culture medium, dimethyl sulfoxide was added, and the resulting mixture was vortexed for 2 to 5 minutes. Absorbance at 490 nm was measured using an enzyme-linked immunosorbent assay reader (M2009PR; Tecan Infinite, Switzerland). All experiments were conducted in triplicate or more.

**Celigo scratch assay**

After resuspending the cells in a complete medium, the logarithmic phase experimental cells were counted after digestion with trypsin. The cells were seeded at a density of 5×10^3^ cells/well. The cells were cultivated in a 100 μL/well, three replicates per group, 37°C, 5% CO_2_ culture condition. The following day, a scratch tool was used to form a small indentation at the centre of the top of each well. At 0 h, the plates were scanned after being gently rinsed twice or thrice with PBS, followed by adding 1% FBS serum-containing medium. After placing the plates in the incubator at 37°C for 24 h at 5% CO_2_, they were scanned with Celigo to detect the amount of migration.

**Cell invasion and migration assays**

For evaluation, the experimental cells in every group were dislodged with trypsin, centrifuged for collection, reconstituted in a serum-free medium, and counted. In the top chamber, cells were injected into a serum-free medium with or without a Matrigel matrix membrane. Cells (5 × 10^5^) were seeded into each well, and 750 μL of 30% FBS culture medium was added to the lower chamber. The steps for developing the cells followed a prescribed pattern. The non-invasive cells were removed from the inner wall of the incubator at 37°C with a moist brush. The outer cell walls were gently washed with PBS, stained with crystal violet, and fixed in 4% paraformaldehyde. The number of cells in each of the nine distinct minute visual areas was measured under a microscope. Each experimental group was tested in triplicate.

**Statistical analysis**

The Mann–Whitney U test was used to compare the LPXN immunological pathways, and *p*-values were adjusted using the Bonferroni technique. The variation in OS across groups was investigated using Kaplan–Meier plots. Using Spearman’s or Pearson’s correlation analyses, we investigated the relationships between LPXN expression levels and stemness, stromal score, drug resistance genes, regulators of immunity, and drug sensitivity. We compared the experimental groups using one-way ANOVA and Bonferroni post-hoc tests. Charts were created using the R programming language (version 4.2.1) and the ggplot2, pheatmap, ggpubr, corrplot, and Survminer applications. A two-tailed *p* < 0.05 was utilised to indicate significance in all experiments.
