# Supplementary material for: An in-depth analysis of the prognostic significance and potential clinical impact of Leupaxin in the immunotherapeutic treatment of esophageal squamous cell carcinoma
Source: Genes Dis. 2025 May 27;13(1):101695. doi: 10.1016/j.gendis.2025.101695 (PMC12624606; doi:10.1016/j.gendis.2025.101695)
Supplement: Supplementary Table 1 — The percentage of positive cells (positive cells/total cells). [file mmc2.doc]

**The percentage of positive cells (positive cells/total cells)**

| **Sample** | **Normal** | | **ESCC** | |
| --- | --- | --- | --- | --- |
| 01 | 84/816 | 10.29% | 1784/1896 | 94.09% |
| 02 | 494/1462 | 33.78% | 1260/1292 | 97.52% |
| 03 | 58/662 | 8.76% | 1071/1103 | 97.10% |
| 04 | 312/1280 | 14.31% | 901/1033 | 87.22% |
| 05 | 48/1110 | 4.32% | 1190/1222 | 97.38% |
| 06 | 680/1712 | 39.71% | 630/1662 | 37.91% |
| 07 | 96/536 | 17.91% | 392/724 | 54.14% |
| 08 | 336/1368 | 24.56% | 560/992 | 56.45% |
| 09 | 78/784 | 9.94% | 1060/1292 | 82.04% |
| 10 | 104/936 | 11.1% | 1470/1502 | 97.87% |

IHC-score = Σpi(i+1) where “pi” represents the percentage of positive cell counts in total cell counts, and the percentage of positive cells was categorized as follows: 0 (less than 5% positive cells), 1 (6-25% positive cells), 2 (26-50% positive cells), 3 (51-75% positive cells), or 4 (more than 75% positive cells). “i” represents the intensity). To evaluate the accuracy of the computer-assisted measurement, the computerized images and the computer-assisted measurements were verified with at least 2 professional pathologist-based scoring results.
