# Supplementary material for: An in-depth analysis of the prognostic significance and potential clinical impact of Leupaxin in the immunotherapeutic treatment of esophageal squamous cell carcinoma
Source: Genes Dis. 2025 May 27;13(1):101695. doi: 10.1016/j.gendis.2025.101695 (PMC12624606; doi:10.1016/j.gendis.2025.101695)
Supplement: Supplementary Table 2 — Clinical information of 32 patients providing tissue samples. [file mmc3.doc]

| Supplementary Table2: Clinical Feature | | | | | | | | |
| --- | --- | --- | --- | --- | --- | --- | --- | --- |
|  | Pathology | Age (year-old) | Sex | Stage | Location of the esophagus relative to the incisors (cm) | Experiment | Treatment regimen | Therapeutic effect |
| 1 | ESCC | 48 | Male | ⅢB | 34-40 | PCR and IHC | Before treatment | - |
| 2 | ESCC | 74 | male | ⅡA | 32-36 | PCR and IHC | Before treatment | - |
| 3 | ESCC | 76 | male | ⅢA | 30-34 | PCR and IHC | Before treatment | - |
| 4 | ESCC | 67 | male | ⅢB | 34-38 | PCR and IHC | Before treatment | - |
| 5 | ESCC | 62 | male | ⅡA | 33-38 | PCR and IHC | Before treatment | - |
| 6 | ESCC | 66 | male | ⅡB | 35-38 | PCR and IHC | Before treatment | - |
| 7 | ESCC | 65 | male | ⅡA | 33-39 | PCR and IHC | Before treatment | - |
| 8 | ESCC | 73 | male | ⅡA | 24-30 | PCR and IHC | Before treatment | - |
| 9 | ESCC | 68 | male | ⅡB | 25-30 | PCR and IHC | Before treatment | - |
| 10 | ESCC | 73 | male | ⅢB | 35-36 | PCR and IHC | Before treatment | - |
| 11 | ESCC | 54 | male | ⅡA | 20-25 | PCR and IHC | Immunotherapy + chemotherapy | Good |
| 12 | ESCC | 60 | male | ⅡA | 27-31 | PCR and IHC | Immunotherapy + chemotherapy | Good |
| 13 | ESCC | 70 | male | ⅠB | 26-30 | PCR and IHC | Immunotherapy + chemotherapy | Good |
| 14 | ESCC | 66 | male | ⅢB | 30-35 | PCR and IHC | Immunotherapy + chemotherapy | Good |
| 15 | ESCC | 58 | male | ⅢB | 33-37 | PCR and IHC | Immunotherapy + chemotherapy | Good |
| 16 | ESCC | 65 | female | ⅠA | 24-27 | PCR and IHC | Immunotherapy + chemotherapy | Good |
| 17 | ESCC | 56 | male | ⅡA | 30-32 | PCR and IHC | Immunotherapy + chemotherapy | Good |
| 18 | ESCC | 54 | female | ⅡA | 34-37 | PCR and IHC | Immunotherapy + chemotherapy | Good |
| 19 | ESCC | 53 | male | ⅡA | 25-30 | PCR and IHC | Immunotherapy + chemotherapy | Good |
| 20 | ESCC | 75 | male | ⅢB | 30-34 | PCR and IHC | Immunotherapy + chemotherapy | Good |
| 21 | ESCC | 57 | male | ⅠA | 24-29 | PCR and IHC | Immunotherapy + chemotherapy | Good |
| 22 | ESCC | 70 | male | ⅡB | 24-28 | PCR and IHC | Immunotherapy + chemotherapy | Good |
| 23 | ESCC | 70 | male | ⅢB | 30-36 | PCR and IHC | Immunotherapy + chemotherapy | Good |
| 24 | ESCC | 51 | male | ⅠB | 23-28 | PCR and IHC | Immunotherapy + chemotherapy | Good |
| 25 | ESCC | 66 | male | ⅣA | 27-30 | PCR and IHC | Immunotherapy + chemotherapy | Poor |
| 26 | ESCC | 65 | male | ⅣA | 28-40 | PCR and IHC | Immunotherapy + chemotherapy | Poor |
| 27 | ESCC | 58 | male | ⅢB | 26-32 | PCR and IHC | Immunotherapy + chemotherapy | Poor |
| 28 | ESCC | 51 | male | ⅣA | 30-40 | PCR and IHC | Immunotherapy + chemotherapy | Poor |
| 29 | ESCC | 66 | male | ⅢB | 30-35 | PCR and IHC | Immunotherapy + chemotherapy | Poor |
| 30 | ESCC | 71 | male | ⅣA | 27-32 | PCR and IHC | Immunotherapy + chemotherapy | Poor |
| 31 | ESCC | 61 | male | ⅢB | 26-30 | PCR and IHC | Immunotherapy + chemotherapy | Poor |
| 32 | ESCC | 54 | male | ⅢB | 30-33 | PCR and IHC | Immunotherapy + chemotherapy | Poor |
